# Men’s and women’s response to treatment and perceptions of outcomes in a randomized controlled trial of injectable opioid assisted treatment for severe opioid use disorder

**DOI:** 10.1186/s13011-017-0110-9

**Published:** 2017-05-19

**Authors:** Heather Palis, Kirsten Marchand, Daphne Guh, Suzanne Brissette, Kurt Lock, Scott MacDonald, Scott Harrison, Aslam H. Anis, Michael Krausz, David C. Marsh, Martin T. Schechter, Eugenia Oviedo-Joekes

**Affiliations:** 1 Centre for Health Evaluation & Outcome Sciences, Providence Health Care, St. Paul’s Hospital, 575- 1081 Burrard St, Vancouver, BC V6Z 1Y6 Canada; 2 School of Population and Public Health, University of British Columbia, 2206 East Mall, Vancouver, BC V6T 1Z3 Canada; 3 Centre de Recherche du Centre Hospitalier de l’Université de Montréal (CRCHUM), Hôpital Saint-Luc, Montréal, QC H2X 3J4 Canada; 4 Providence Crosstown Clinic, Providence Health Care, 84 West Hastings Street, Vancouver, BC V6B 1G6 Canada; 5 Department of Psychiatry, Faculty of Medicine, Detwiller Pavilion 2255 Wesbrook Mall, Vancouver, BC V6T 2A1 Canada; 6 Northern Ontario School of Medicine, 935 Ramsey Lake Road, Sudbury, ON P3E 2C6 Canada

**Keywords:** Opioid-dependence, Gender, Clinical trial, Patient perceptions

## Abstract

**Background:**

To test whether there are gender differences in treatment outcomes among patients receiving injectable opioids for the treatment of long-term opioid-dependence. The study additionally explores whether men and women have different perceptions of treatment effectiveness.

**Methods:**

This study is a secondary analysis from SALOME, a double-blind, phase III, randomized controlled trial testing the non-inferiorirty of injectable hydromorphone to injectable diacetylmorphine among 202 long-term street opioid injectors in Vancouver (Canada). Given this was a secondary analysis, no a priori power calaculation was conducted. Differences in baseline characteristics and six-month treatment outcomes (illicit heroin use, opioid use, crack cocaine use, non-legal activities, physical and psychological health scores, urine positive for street heroin markers, and retention) were analysed by gender using fitted models. Responses to an open ended question on reasons for treatment effectiveness were explored with a thematic analysis.

**Results:**

Men and women differed significantly on a number of characteristics at baseline. For example, women were significantly younger, presented to treatment with significantly higher rates of prior month sex work (31.5% vs. 0%), and used significantly more crack cocaine (14.71 vs. 8.38 days). After six-months of treatment there were no significant differences in treatment outcomes by gender, after adjusting for baseline values. For both men and women, improved health and quality of life were the most common reasons provided for treatment effectiveness, however women were more specific in the types of health improvements.

**Conclusions:**

Despite presenting to treatment with vulnerabilities not faced to the same extent by men, at six-months women did not differ significantly from men in tested trial efficacy outcomes. While the primary outcome in the trial was the reduction of illicit opioid use, in the open-ended responses both men and women focused their comments on improvement in health and quality of life as reasons for treatment effectiveness. The supervised model of care with injectable medications provides a particularly suitable framework for providing care to opioid-dependent men and women not attracted or retained by other treatments. The absence of statistical differences reported in this secondary analysis may be due to lack of adequate statistical power to detect meaningful effects.

**Trial registration:**

This trial is registered with ClinicalTrials.gov (NCT01447212) Registered: October 4, 2011 at the following link: https://clinicaltrials.gov/ct2/show/NCT01447212.

## Background

Opioid-use disorder is a chronic relapsing condition characterized by patterns of continued illicit drug use, periods of treatment, abstinence from street opioids, and relapse, with a number of associated harms to the individual and to the community [1, 2]. Data on opioid use disorder has shown this chronic condition to be more prevalent among men than among women, and as such, conclusions derived primarily from the experiences of men are often extrapolated to women [3, 4]. Studies of opioid-dependence consistently suggest that there are few (although inconsistent) or no gender differences in treatment outcomes [5–9]. Nevertheless, in studies of opioid use disorder, women are consistently found to be faced with elevated exposure to social and medical vulnerabilities associated with their substance use, suffering more severe emotional and physical symptoms as compared to men [6, 10–12].

The concept of gender refers to the socially constructed bounds of being a “female” or a “male” in a society, and is linked to the social and political context [13, 14]. These socially constructed conceptions of gender and gender roles influence human interactions, behaviours, and conceptions of the physical body, ultimately producing gender identities [14]. Gender based analyses aim to assess potential differences in treatment outcomes for men and women, and to identify the possible impact of policies and programs in relation to these differences [14].

Studies that have investigated gender differences in substance use treatment outcomes such as retention suggest few or no differences [6, 15]. However, gender based analysis of outcomes among patients receiving injectable treatments are limited, with only two studies [9, 16]. For example, the North American Opiate Medication Initiative (NAOMI) clinical trial compared supervised injectable diacetylmorphine (pharmaceutical grade heroin) (DAM) with oral methadone maintenance treatment (MMT) among long-term opioid injectors in Vancouver and Montreal (Canada). In NAOMI, overall treatment retention rates did not differ for women (64.8%) and men (75.4%). Further, among participants receiving DAM there were no significant gender differences in drug use or illegal activities [17]. These findings indicated that among long-term opioid dependent women not benefiting from available treatments, DAM was more effective than MMT.

It is pivotal to ground a discussion of men’s and women’s experiences of injectable opioid assisted treatment in the broader literature on structural vulnerability. Structural vulnerability refers to an individual’s condition of being at risk of negative outcomes, through their position in relation to socioeconomic, political, and cultural normative hierarchies [18, 19]. Women present to opioid assisted treatment with a number of vulnerabilities, for example, lower rates of education, more prevalent histories of sexual abuse, higher rates of infectious disease, higher suicide rates, higher rates of crack cocaine use, sex work, and mental distress [4, 9, 16, 20]. These vulnerabilities are a product of a number of attributes, including gender [21].

The present study was conducted with a sample of long-term opioid dependent women and men who were enrolled in a unique model of care, receiving injectable opioid assisted treatment. Under this model of care, participants received injectable medications under the supervision of registered nurses. This daily contact allows the opportunity for comprehensive care, attending to the many complex needs of the patients, thereby optimizing the provision of services that are in line with the construct of patient centered care, where the needs of the patients are prioritized. A patient centered approach allows for the social positions of patients to be recognized, and for the promotion of cultural safety, both of which are more recent dimensions of patient centered care being considered in the delivery of services for marginalized groups [22–24]. Such an approach accounts for components of a patient’s position, including race, gender, sexuality, and socioeconomic status, so that treatments are responsive to the unique circumstances with which patients experience care [25].

In most clinical trials, patient outcome assessments are developed by clinicians and researchers and tend to neglect the perspectives of patients [26]. This is of concern, given the evidence suggesting that among patients receiving treatments for substance dependence, conceptions of treatment effectiveness may differ greatly from those of clinicians and researchers [27, 28]. As such, there has been an increasing interest in collecting patient perceptions of treatment outcomes. The present study aims to 1) determine whether men and women differ in their response to treatment and; 2) to explore men’s and women’s perceptions of treatment effectiveness.

## Methods

### Setting, participants, study design

The Study to Assess Longer Term Opioid Medication Effectiveness (SALOME) was a phase III, double blind clinical trial testing the non-inferiority of hydromorphone (HDM) to diacetylmorphine (DAM) for the treatment of long-term opioid-dependence. Long-term injection opioid users who were not sufficiently benefitting from available treatments (i.e. continuing to inject illicit opioids) were recruited from the Lower Mainland of British Columbia, with most residing in the Downtown Eastside of Vancouver. Applicants were excluded if they were pregnant, or planning on becoming pregnant, had an imminent period of extended incarceration, or had severe medical conditions contraindicated for treatment with DAM or HDM (e.g. respiratory problems).

Participants were randomly assigned to receive injectable diacetylmorphine (*n* = 102) or hydromorphone (*n* = 100) for six months. These medications were delivered under a “supervised model of care”, whereby registered nurses supervised participants’ self-administration of injectable opioids at the study site up to three times per day. The supervised model of care operates with three main objectives: 1) keeping patients and the community safe by providing pharmaceutical-grade injectable opioids under supervision (e.g. for prompt response in case of an overdose) and keeping the medications onsite (to minimize the potential risk to others); 2) Building relationships with patients that foster trust through daily interactions; and 3) providing opportunities for comprehensive care. In SALOME, medications were provided at up to 400 mg per dose, and up to 1000 mg per day [29]. Participants could adjust the dose and frequency of sessions in consultation with their physician. Doses were titrated individually in order to achieve a safe and effective dose for each participant. Doses were determined over a 3-day titration phase by the participant and the nurse. In rare cases a slower titration process was used based on the participant prior clinical experience or medical history [29]. In consultation with the study physician, participants could add oral methadone to their care at any time. Additional details on the participant profile, screening procedures, study design and main results have been published elsewhere [29–31].

### Study measures

At baseline, prior to randomization, participants completed questions on a series of standardized instruments on topics such as drug use, and physical and psychological health [32, 33]. Treatment outcomes were self-reported with the exception of urinalyses, and were collected at 6 months. Outcomes assessed included days of illicit heroin use, days of any illicit opioid use, days of non-legal activities, days of crack cocaine use, the proportion of urine samples positive for street heroin markers, the proportion of participants receiving treatment 28 days or more in the prior month (retention), and measures of physical and psychological health collected with the Maudsley Addiction Profile [32]. Adverse events were recorded as described in further detail in the main SALOME trial results [29] and classified with MedDRA codes. Given this is a secondary analysis from a non-inferiority clinical trial testing two medications in double blind conditions, there was no a priori power calculation for the present analysis testing differences in outcomes by gender.

Data on perceptions of why the treatment is effective come from one interviewer-administered questionnaire conducted in reference to the first 6 months of treatment. As part of the questionnaire assessing blinding, participants were first asked: “Do you think this treatment was effective?” Participants could respond, “yes”, “no”, or “unsure”. Participants then provided open-ended responses to the question “why do you think this treatment was/was not effective?” In a clinical trial, protection of the blinding, and the collection of primary outcome data are prioritized [34]. Therefore, as to not interfere with the clinical trial findings participants were not prompted to provide an in-depth response. Instead, the interviewer posed the question and then recorded the participant’s verbatim response. Responses ranged from a few words to a few sentences. While this does not provide in-depth qualitative data, it provides novel information, and a perspective on treatment effectiveness not typically captured in the context of stringent randomized controlled trials.

### Statistical analyses

Baseline characteristics were tested by gender using Chi-Square tests and Student’s t tests. At six months, differences between men and women were tested on eight outcome variables. Outcome variables were continuous (number of days in the prior month for illicit heroin use, any illicit opioid use, involvement in non-legal activity, crack cocaine use; MAP physical health score, and MAP psychological health score) and as proportions (urine positive for street heroin markers, and at least 28 days retention in treatment in the prior 30 days).

A separate model was fitted for each outcome, as dependent variables, for the differences between women and men. At six-months, there were a high proportion of participants reporting zero days in reference to prior 30 day illicit heroin use, illicit opioid use, non-legal activity, and crack cocaine use. As such, zero inflated poisson regression was used for these outcomes, given this model allows for the excess zeros, which cannot be predicted by the standard poisson model [35]. These data are presented as the mean difference (with confidence interval) in the number of days at six-months. Linear regression was performed for the physical and psychological health variables, showing mean difference with confidence intervals. Finally, odd ratios were calculated using a logistic regression for the urine positive markers and retention variables.

All models were adjusted for treatment arm and the average daily dose. Models were also adjusted for baseline values using analysis of covariance (ANCOVA) to account for gender difference at baseline [36]. This is with the exception of the retention variable, and urine positive for street heroin markers (there is no baseline value for retention, and as per inclusion criteria all patients had urine positive for street opioids at baseline). For zero-inflated Poisson models, we compared the model fit with ordinary Poisson regression. For the logistic regression model and multiple regression models, goodness-of-fit was assessed by comparing to the null model. Residuals were also examined to identify potential outliers or misspecified models.

There were 4 missed assessments at 6 months (2 deceased, 1 missed visit, and 1 lost to follow-up). Missing values were imputed with multiple imputation, except when data were missing due to death, thus avoiding assigning a score to a deceased participant [37].

Types of non-legal activities and sources of income, for men and women at baseline and at six-months were explored using descriptive analysis. At each time point, differences between men and women in prior month legal, non-legal, and total sources of income were tested using Wilcoxon Rank Sum Tests. Differences in the proportion and days of non-legal activities were tested by gender using Chi-Square tests and Student’s *t* tests. These descriptive data are presented in Tables 3 and 4. All statistical analyses were conducted using SAS 9.4 [38]. Two-sided tests were used with a significance level of 0.05.

### Thematic analysis of open-ended comments

Thematic analysis of participant perceptions of treatment effectiveness was conducted. Two of the authors independently coded the transcripts (HP and KM). An inductive approach was taken with each comment assigned a code based on its semantic content. Themes and relationships across all participants’ comments were developed using the strategy of constant comparison [39]. After initial independent coding, the authors (HP and KM) met to discuss and review the initial list of themes. Themes were then further refined to ensure congruency between the content and the assigned theme (HP, KM, and EOJ). Analysis of open-ended comments was conducted using NVivo software [40].

Women’s and men’s perceptions of treatment effectiveness were analysed together, rather than separately. Coders were blinded as to the gender of participants. Once the stages of coding were complete the number of women and number of men making a reference to each theme was recorded. It is possible that one participant made more than one reference at a given theme. As such, data presented are the number of participants that have at least one reference coded in a given category. This ensures themes are not overinflated by one particular participant’s references, and allows for the comparison of the total number of women compared to men that made a reference.

## Results

Of the 202 SALOME participants, 62 self-identified as women (including 3 transgender participants identifying as women), and 140 self-identified as men. At baseline, men and women differed on various socio-demographic, health, drug use, and treatment variables. Women were significantly younger than men . There were a significantly higher proportion of women self-identifying as Indigenous, and a significantly higher proportion of women compared to men that had ever been paid in exchange for sex. Men reported significantly more months incarcerated in lifetime. Women reported significantly higher symptoms on the Maudsley Addiction Profile (MAP) physical and psychological health scores, indicating poorer baseline health compared to men. There were nearly double the proportion of women with HIV compared to men, and women reported smoking significantly more days of crack cocaine in the prior month compared to men. Men and women reported similar patterns of prior month heroin and illicit opioid use (Table 1).

**Table 1:** Baseline socio-demographic, health, drug use and treatment profile of SALOME participants by gender

	Total *N* = 202	Women *N* = 62	Men *N* = 140
Socio-demographics			
Age*	44.33 ± 9.63	40.66 ± 9.34	45.95 ± 9.34
Currently has an intimate partner*	74 (36.63)	32 (51.61)	42 (30.00)
Self- identify as Indigenous^a^*	62 (30.69)	29 (46.77)	33 (23.57)
High school certificate or higher	108 (53.47)	31 (50.00)	77 (55.00)
Any non-stable housing in prior 3 years^b^	141 (69.80)	38 (61.29)	103 (73.57)
Paid in exchange for sex in the prior month	20(9.90)	19 (30.65)	0(0)
Ever paid in exchange for sex*	83 (41.09)	52 (83.87)	31 (22.14)
Months ever incarcerated*^c^	10 [1–36]	2 [0–6]	18 [3–60]
Days of non-legal activities in prior month^d^	14.15 ± 13.71	14.98 ± 13.59	13.79 ± 13.79
Health			
HIV Positive*	30 (14.85)	14 (22.58)	16 (11.43)
MAP Physical Health Score^e^*	12.17 ± 8.01	14.92 ± 9.12	10.92 ± 7.15
MAP Psychological Health Score^e^*	9.40 ± 8.97	12.35 ± 10.56	8.05 ± 7.83
Drug Use and Treatment			
Days using any illicit opioids	27.95 ± 4.19	28.15 ± 4.48	27.86 ± 4.07
Heroin, injection	25.38 ± 7.99	25.84 ± 7.69	25.18 ± 8.14
Times of heroin use on a typical day	3.40 ± 2.59	3.73 ± 2.50	3.26 ± 2.62
Crack cocaine, smoked*	10.32 ± 12.72	14.71 ± 13.62	8.38 ± 11.83
Times attempted MMT in prior 5 years^f^	2.81 ± 2.09	3.06 ± 2.10	2.69 ± 2.09

*Indicates significance below *p* < 0.05

Plus minus values (±) indicate mean and standard deviation; Values in parentheses indicate number (n) and percentage (%); Values in brackets represent median and interquartile range (Q_1_–Q_3_). Statistics are *p* values for a t-test or chi-square test:

*SD* Standard Deviation, *MAP* Maudsley Addiction Profile, *HIV* Human Immunodeficiency Virus, *MMT* Methadone Maintenance Treatment

^a^Indigenous Ancestry refers to the self-report of any Inuit, Metis or, First Nations Ancestry

^b^Non-stable housing refers to living in single resident occupancy hotel rooms with restrictions, couch surfing, outdoors, vehicles, or in public places

^c^Data are zero saturated and heavily skewed. As such, median and interquartile ranges are presented

^d^Days of non-legal activities is a measured as a sum of the number of days in the prior month engaged in any of: dealing of drugs, property theft, violence, disorderly conduct, sex work, major driving violations, and broken conditions imposed by the legal system

^e^MAP scores range from 0 to 40; higher scores indicate poorer physical or psychological health

^f^Data come from PhamaNet, British Columbia’s Provincial pharmacy dispensation database

Regarding 6-month treatment outcomes, after adjusting for baseline values, dose, and treatment arm, there were no differences found between men and women, with the exception of psychological health, where women’s health at 6 months was significantly better than that of men: mean difference: -2.39 (95% CI: -4.72, −0.07) (Table 2).

**Table 2:** Treatment outcomes by gender at six months

Outcomes at six months	Women *N* = 62	Men *N* = 140	Women vs. Men *N* = 202
Street opioid use			
Days illicit heroin use^a^	3.69 (2.08, 5.52)	3.84 (2.54, 5.27)	−0.15 (−2.16, 1.98)
Days illicit opioid use^a^	5.28 (3.18, 7.59)	4.84 (3.43, 6.26)	0.44 (−1.93, 3.17)
Proportion of urine positive for street heroin markers^c^	0.27 (0.18, 0.40)	0.25 (0.16, 0.35)	OR 1.16 (0.53, 2.51)
Retention in treatment			
Proportion of participants receiving treatment ≥28 days^c^	0.83 (0.72, 0.91)	0.79 (0.71, 0.85)	OR 1.34 (0.62, 2.87)
MAP health symptom scores			
Physical health^b^	12.29 (10.43, 14.15)	11.37 (10.07, 12.67)	0.92 (−1.38, 3.23)
Psychological health*^b^	6.95 (5.01, 8.90)	9.35 (8.01, 10.69)	−2.39 (−4.72, −0.07)
Other outcomes			
Days of non-legal activity^a^	3.61 (1.61, 5.90)	3.14 (1.86, 4.53)	0.47 (−1.82, 3.14)
Days of crack cocaine use^a^	7.10 (4.70, 9.76)	5.16 (3.26, 7.62)	1.95 (−0.08, 4.28)

*Indicates significance below *p* < 0.05

Differences in proportions (urine positive and retention) are presented as odds ratios (OR). For all other variables mean difference in days or scores are presented. Both proportions and means are presented with 95% confidence intervals in brackets

All models were adjusted for treatment arm and the average daily dose. Models were also adjusted for baseline values using analysis of covariance (ANCOVA) to account for gender difference at baseline. This is with the exception of the retention variable, and urine positive for street heroin markers (there is no baseline value for retention, and as per inclusion criteria all patients had urine positive for street opioids at baseline)

^a^Continuous outcomes with an excess of zero counts: Zero-inflated Poisson regression was used. Adjusted mean difference between the two gender groups and confidence intervals were estimated by the Bootstrap method

^b^Continuous outcomes: Linear regression models were used to estimate the mean difference and 95% CI

^c^Binary outcomes: Logistic regression was used to estimate odds ratios and 95% CIs to compare the proportions between groups

There were no significant differences between men and women in in the number of related adverse events or proportion of participants with at least one related event in the DAM arm, nor in the HDM arm. The same results were found regarding related immediate post-injection reaction or injection site pruritus, somnolence, serious adverse events and opioid overdoses that required the use of naloxone. In the DAM arm, women had a lower dose than men. None of the potential explanatory factors tested were significant, nor provided explanation for the difference between men and women in psychological health (i.e. differences persisted even after adjusting for baseline values, dose, and treatment arm, and exploring adverse events by gender).

There were no significant differences between men and women in prior month income from non-legal, legal, or total sources at baseline nor at six-months (Table 3). Among both men and women, over 70% of legal income at baseline and at six-months came from income assistance. Days of involvement in sex work, drug dealing and property theft are presented in Table 4. There were a higher proportion of men involved in drug dealing compared to women, both at baseline and at six-months. At baseline, 90.00% of women and 87.50% of men that engaged in drug dealing, did so to support their own use, rather than for income. The proportion of men and women engaged in property theft were 15.71% and 17.74% respectively at baseline and 5.71% and 4.84% at six months. The proportion of participants involved in sex work was significantly different between men and women at baseline (0% vs. 30.65%) and six months (1.43% vs. 20.97%).

**Table 3:** Prior Month Income from Legal, Non-Legal, and Total Sources by gender

Source of income	Women *N* = 62		Men *N* = 140	
	N(%)	Median (IQR)	N(%)	Median (IQR)
Baseline				
Non-legal	45 (72.58)	2000.00 (800.00, 4600.00)	86 (61.43)	1750.00 (500.00, 4000.00)
Legal	61 (98.39)	1111.00 (850.00, 1400.00)	140 (100.00)	1150.00 (900.00, 1587.50)
Total	62 (100.00)	2282.50 (1400.00, 4200.00)	140 (100.00)	2115.50 (1487.50, 4022.00)
Six-months				
Non-legal	23 (37.10)	1000.00 (500.00, 1500.00)	38 (27.94)	1500.00 (250.00, 3000.00)
Legal	60 (96.77)	1080.00 (836.00, 1300.00)	135 (96.43)	1100.00 (906.00, 1330.00)
Total	62 (100.00)	1300.00 (960.00, 2120.00)	136 (100.00)	1200.00 (957.50, 1955.00)

Medians are in Canadian Dollars. IQR = Interquartile Range. Medians and IQR data is presented for women and men reporting at least one dollar of income in each source

Legal Sources: Employment, Income Assistance, Pension, Disability, Money from partner, family or friends

Non-legal Sources: Drug dealing, property theft, and sex work

There were no significant differences found between men and women in regards to baseline or six-month non-legal, legal, or total income

**Table 4:** Self-reported involvement in non-legal activities by gender at baseline and six-months

	Women *N* = 62		Men *N* = 140	
Type of Activity	N(%)^a^	Days in prior 30^b^	N(%)^a^	Days in prior 30^b^
Baseline				
Drug Dealing	23 (37.10)	22.04 ± 10.42	67 (47.86)	21.10 ± 11.10
Property Theft	11 (17.74)	15.27 ± 13.09	22 (15.71)	18.64 ± 12.30
Sex Work	19 (30.65)*	14.21 ± 10.13	0 (0)	0
Six-months				
Drug Dealing	10 (16.13)	15.00 ± 11.68	27 (19.29)	17.04 ± 11.64
Property Theft	3 (4.84)	14.67 ± 6.11	8 (5.71)	7.75 ± 9.56
Sex Work	13 (20.97)*	11.46 ± 10.49	2 (1.43)	11.00 ± 1.41

*Indicates significance below *p* < 0.05 Plus minus values (±) indicate mean and standard deviation; Values in parentheses indicate number (n) and percentage (%);

Column presents the proportion of women or men reporting one or more days in the prior 30 days engaging in each of the corresponding listed activities

^a^Column presents the average number of days in the prior 30 days engaged in each of the corresponding listed activities, among those reporting at least 1 day in column (a)

^b^Column presents the proportion of men and proportion of women reporting one or more days in the prior 30 days engaging in each of the corresponding listed activities

Of the 202 participants, 191 participants (132 men, and 59 women) gave a response as to why they thought the treatment was or was not effective (yes, no, or unsure). Among the 11 missing respondents, 4 did not complete the 6-month interview, and 7 did not complete this question. Among those seven participants that did not complete this question, three were women and four were men. In response to the question of whether the treatment was effective these participants responded; yes (*n* = 4); unsure (*n* = 1); missing (*n* = 2).

Among all 191 respondents, 175 (91.62%) thought the treatment was effective, 10 (5.23%) thought the treatment was not effective, and 6 (3.14%) were unsure. A total of 436 references were coded among 191 participants. Each of the participants that thought the treatment was not effective (*n* = 10) gave one of the following reasons: negative side effects, withdrawal symptoms, or craving. Each of the participants that were unsure gave statements about both negative (e.g. side effects) and positive aspects (e.g. reduced street use) of the treatment (*n* = 6).

Seven themes emerged for reasons participants perceived the treatment to be effective (Table 5). The most commonly referenced themes were improved health and improved quality of life, followed by stopped or reduced street drug use and stopped or reduced non-legal activities. Participants also discussed reduced craving or withdrawal, spending money on things other than drugs, and aspects of the model of care in which the treatments are delivered.

**Table 5:** Participant reasons for Treatment Effectiveness

Themes	Total *N* = 191 N (%)	Women *N* = 59 N (%)	Men *N* = 132 N (%)
Improved Health	79 (41.36)	23 (38.98)	56 (42.42)
Improved Quality of Life	64 (33.5)	19 (32.20)	45 (34.09)
Stopped or reduced street use	57 (29.84)	16 (27.12)	41 (31.06)
Stopped or reduced non-legal activity	41 (21.47)	8 (13.56)	33 (25.00)
Reduced craving or withdrawal	39 (20.42)	14 (23.73)	25 (18.94)
Spending money on things other than drugs	24 (12.57)	7 (11.86)	17 (12.88)
Model of Care	24 (11.88)	5 (8.47)	19 (14.39)

Responses arise from an open-ended questions asked of participants after 6 months of treatment

Themes are listed in order of frequency

Responses are in reference to the treatment participants thought they were receiving after the first 6 months of treatment (treatment was blinded)

Columns refer to the number of participants (total, women, men) that made a reference at a given theme and the percent referencing a given theme out of all participants (total, women, men) that provided a response. (e.g. for Improved Health: Total = 79/191 = 41.36%; Women = 23/59 = 39.98%; Men = 56/132 = 42.42%)

Many participants made statements about the multiple components of their health and quality of life that were bettered by the treatment. Men gave more general statements such as “*My health is better”,* while women generally explained in more depth what better health meant for them:

*“Everything in my life has changed, I have housing now, I eat every day, I'm sleeping better, I am way healthier, less stressed. I have a cat now that I spayed and vaccinated and take care of - I haven't had a pet in 10 years because I was too all over the place mentally. I also have regained my relationship with my mom, my siblings, my kids, my partner. I was so wrapped up in addiction that I became a non-person. Now I'm woken up. I'm back.” (Participant 6089, Gender: Woman)*

Men and women similarly referenced general improvements in lifestyle and social life as components of quality of life. Men however were more likely to reference not having to “hustle” (“*Nice not to have to get up and run the race every day*”) and women were more likely to reference not having to do sex work (“*Before, I worked (sex work)… now I don't have to do it anymore”*). Many of the statements made touched on multiple components of health and lifestyle:

*“I am very content with what I'm getting and the whole program. It's given me time to reflect on things I was too busy to reflect on before. Being wired is a full time job. Getting the coin, scoring, enjoying the high: it consumes a big part of your day. Now, I have more time, and I'm finding more things to do that I like, like cycling. I'm helping one of the other participants become a better reader. Hanging out with friends, playing pool.” (Participant 6125, Gender: Man)*

Men’s descriptions tended to include statements about reducing or stopping criminal involvement. Men specified reduced theft, dealing, and worries of going to jail, while only two women made such specific references. Women and men similarly referenced stopping or reducing street drug use, mostly referring to opioid use.

*“I'm taking care of business now, getting my life on track, of course my money situation has improved, healthier now, I was doing crime before, and now there's no need for money, for doing whatever to get money, I have a choice now, and I choose to do hardly any crime”. (Participant 6201, Gender: Man)*

*“I am not needing to score as often as I used to… I am less desperate.” (Participant 6046, Gender: Transgender Woman)*

*“I had no craving, no desire to go out and use, I have a chance at a normal life. Salome is crazy good! It works.” (Participant 6020, Gender: Woman)*

*“It is the first time I have been totally clean of street heroin. It really feels like the difference between life and death.”(Participant 6065, Gender: Man)*

Both women and men referenced the fact that the medication reduces cravings and symptoms of withdrawal as a reason for treatment effectiveness. While men and women referenced improved financial situation, men tended to make more comments on spending money on things other than drugs as compared to women. Comments made included statements like: “*I still have money at the end of the month*”, or “*I own things again, I am not spending it all on dope.” and “Hardly spend money on drugs, spend it all on food”.*

Men and women had similar descriptions surrounding the model of care as a reason for treatment effectiveness. These comments reflect that the model allowed for individualized care, that it provided structure and security, and it is less stigmatizing than other models.

*“I haven't used street heroin since study started; I am on injectable and that's a big part of the addiction. [The program] offers a routine, a security blanket, don't need to worry anymore. It's changed my whole thinking” (Participant 6028, Gender: Man).*

## Discussion

Findings of the present study were consistent with the existing literature on treatment outcomes among men and women receiving injectable opioid assisted treatments for severe opioid use disorder [8, 9]. Overall, no significant differences were found by gender in the tested efficacy outcomes. This finding is particularly relevant considering women presented to treatment with vulnerabilities not faced to the same extent by men. The supervised model of care with medically-prescribed injectable opioids aims to reach and treat individuals that, despite other options being available, continue injecting opioids in the street. The study participants presented to treatment after many years of injecting street opioids, with more than half of them reporting having a chronic condition that interferes with their daily lives, as well as histories of incarceration, sex work, and homelessness [31]. While SALOME participants represent the most vulnerable population of long-term street opioid-injection users, women face particular vulnerabilities and as such, it is important to determine whether this treatment engages and retains them into treatment as well as men.

After six months of treatment, no significant differences between men and women were found in days of illicit heroin use, illicit opioid use, nor proportion of urine positive for street heroin markers. A similar proportion of men and women were retained at six-months (approximately 80% had received treatment at least 28 days in the prior 30). Men and women reported a similar number of days using crack cocaine and involved in non-legal activity. While there were no significant differences in physical health scores, women’s psychological health scores were significantly better than men’s, after adjusting for baseline values, dose, and treatment arm. This is similar to findings in the NAOMI trial, where women in the DAM arm had significantly better psychological health scores than women in the methadone maintenance treatment arm, yet this difference was not seen in men [17].

While men and women did not differ in terms of days of overall involvement in non-legal activities, there were gender differences when looking into the specific types of activities. For example, at both baseline and six-months significantly more women were involved in sex work as compared to men. Interestingly, the proportion of women engaged in sex work was not reduced significantly from baseline to six-months. While we are limited in our analysis and conclusions due to the small sample size, continued involvement in sex work presents a particular vulnerability, with potential implications for patterns of street drug use and health outcomes. In a prior clinical trial with a similar population, women engaged in sex work were more likely to continue injecting in the street and using cocaine. Also, women who were retained were less likely to be involved in sex work [41].

With such a high retention rate at six months for women receiving injectable treatment in SALOME (83%), there is a compelling case to make to offer this treatment to women that continue injecting in the street despite other treatment options being available. Access to injectable medications can provide a starting point to reconnect with the health care system, and work with patients to meet their individual needs. The supervised model of care is a bundle, meaning that every participant is offered all services available at the clinic. Participants are connected with services inside and outside of the clinic, based on their needs and willingness. Engaging with the clinic services up to three times a day for the medication provides nurses and health care allies opportunities to engage with the patients, to meet them where they are at, and to respond to particular vulnerabilities faced by patients in a timely manner.

This study also investigated participants’ reasons for treatment effectiveness. Prior studies suggest that distinct perceptions of treatment outcomes and effectiveness can be determined with gender-specific analyses. For example, a study of treatment outcomes among offenders in a residential substance use treatment program showed that men and women had differing perceptions of treatment, and emphasized the importance of considering perceptions of outcomes to best understand treatment needs [42]. Further, among a sample of long-term opioid dependent men and women in Vancouver, Canada, it was found that despite reporting similar treatment satisfaction scores, men and women had distinct perceptions of the positive aspects of treatment [43]. This evidence supports the added value of including open-ended questions to complement quantitative outcomes in studies of treatment effectiveness.

Our study found seven major themes. A similar proportion of men and women made references to each of these themes. There were however, particular nuances in the way in which men and women spoke about each of the themes. For example, women’s comments surrounding improvements in health and quality of life were much more descriptive than men’s. Women’s comments ranged from references to personal growth and stability, to physical well-being and improvements in their social lives. Better health for women meant rebuilding relationships with family members, stronger self-connection, and better self-care in ways in which they saw tangible improvements (e.g. better nutrition leading to weight gain). These descriptions suggest that this treatment is particularly impactful for women’s health.

Men made comments about reducing or stopping non-legal activities while receiving the treatment. A significant reduction in non-legal activities is a consistent outcome among studies of injectable opioid assisted treatment [29, 44, 45]. This positive outcome is evident in the men’s comments surrounding reductions in crime, describing reduced worry about arrest, no longer having the difficulty of engaging in the daily “hustle” to attain drugs, and freeing time in their day to engage in other meaningful activities. Although men and women had no differences in the reduction in the number of days engaged in non-legal activities, this study shows this outcome to be particularly relevant for men, according to their comments.

Before starting treatment with injectable opioids, it is possible that the medication itself (i.e. pharmaceutical grade heroin) is the most attractive component of care for the participant [46]. In a prior clinical trial it was found that many participants were drawn to the treatment just because of the possibility of receiving pharmaceutical grade heroin [47]. However, in the present study, when participants were asked why the treatment was effective for them, there was not a strong emphasis on the medication itself as a reason for treatment effectiveness. When participants made comments about the medication they were general (e.g. “I am not dope sick” or “no withdrawal”). Instead, statements surrounding treatment effectiveness were primarily derived from comments about improvements made in various aspects of the participants’ lives that have been positively impacted by the treatment and services they had received. These descriptions were about health and quality of life, areas that participants were able to focus on given access to an effective medication.

These findings are consistent with those of The Experimental Narcotics Prescription Programme in Andalusia (PEPSA), a clinical trial of heroin-assisted treatment in Spain. This study found that when heroin, a substance typically obtained non-legally was medically prescribed there was a shift in the significance given to the substance itself [48]. Once the program took care of the injectable medication, many other needs beyond the medication became evident [49] allowing for participants to focus on improvements in physical and mental health, family relations, and employment.

In the present study, the men’s and women’s accounts of reasons for treatment effectiveness are in line with the idea that once participants were provided with access to the medication they were dependent on (i.e. physical treatment needs were met), they regained meaningful space and time to focus on other aspects of their lives. The recognition that patients’ needs may broaden throughout the course of treatment suggests that the treatment system must be prepared to respond to these needs, in a way that is flexible and reactive to the patient and their progress. Prior studies on patients receiving injectable opioids under supervision suggest that engaging patients in research regarding their perceptions of treatment (e.g. treatment expectations, treatment effectiveness) can provide a comprehensive assessment of treatment challenges and treatment needs in order to optimize the treatment received as they progress through treatment [50, 51].

In the present study, participants were asked to share their perceptions of treatment effectiveness. Evidence suggests that providing patients with the opportunity to discuss and share their perceptions of their treatment outcomes and effectiveness can serve as a source of empowerment [52, 53]. This is particularly relevant to shifting away from traditional paternalistic approaches to the provision of health care services, to a system more focused on patient autonomy, shared decision making and patient satisfaction [54]. Involving patients in the stages of outcomes research can work to enrich the scope with which researchers assess outcomes, can promote patient knowledge, and can contribute to an informed dialogue between patients and their health care providers [54]. Future clinical trials should consider the integration of similar open-ended questions on patient perceptions of treatment effectiveness to achieve these benefits.

There are several limitations that must be recognized. Given the nature of the study design, we are limited in our ability to observe more gender specific aspects of treatment beyond that of efficacy. Although we found that women in the DAM arm used a daily and average dose significantly lower than men, none of the potential explanatory factors tested were significant, nor provided explanation for this difference. It is important to note that doses were individually titrated, that overall there were no gender differences in treatment outcomes and that women were not more likely than men to correctly guess the treatment assignment [34]. As such, it is possible that there is some unobserved variable driving this difference in dose, or that the sample size is too small to detect an effect. Data on participant perceptions of treatment effectiveness were collected while participants continued to receive blinded medication. This limited the depth of qualitative data that could be collected on perceptions of treatment effectiveness, as the collection of primary outcome data and protection of the blinding had to be prioritized. Finally, three participants self-identified as transgender women, and were included in the analysis as women. We recognize that transgender people face unique structural vulnerabilities, however with such a small sample we were not able to conduct separate meaningful analysis. The present study is a secondary analysis of data from the SALOME clinical trial. No a priori power calculations were conducted before performing the analyses reported herein. Hence, the analyses presented may be statistically under powered to detect clinically meaningful differences.

## Conclusions

The supervised model of care with injectable medications provides a particularly suitable framework for providing care to opioid-dependent men and women not attracted or retained by other treatments. Regardless of presenting to treatment with particular vulnerabilities not reported to the same extent by men, women achieved similar outcomes. As such, there is an important case to be made to make this treatment available to women. In the present clinical trial treatment effectiveness was meaningfully explained when participant perceptions were accounted for. Descriptions were centered on health and quality of life, areas that participants were able to focus on given access to a reliable and stable effective medication. Building on prior findings, this study adds evidence to support the provision of supervised injectable diacetylmorphine or hydromorphone to the most vulnerable men and women that inject street opiates and are not attracted or reached by other treatments.

## Abbreviations

DAM: Diacetylmorphine
HDM: Hydromorphone
HIV: Human immunodeficiency virus
MAP: Maudsley addiction profile
MMT: Methadone maintenance therapy
SALOME: Study to assess longer term opioid medication effectiveness
SD: Standard deviation
